# Immunomodulatory Effects of Modified Bovine Colostrum, Whey, and Their Combination with Other Natural Products: Effects on Human Peripheral Blood Mononuclear Cells

**DOI:** 10.1016/j.curtheres.2023.100720

**Published:** 2023-10-05

**Authors:** Xuesheng Han, David Vollmer, Elena Y. Enioutina

**Affiliations:** 1 4Life Research USA, Sandy, Utah; 2 Division of Clinical Pharmacology, Department of Pediatrics, University of Utah School of Medicine, Salt Lake City, Utah

**Keywords:** Bovine colostrum filtrate, Cytokines, Egg yolk extract, Human peripheral blood mononuclear cells, whey

## Abstract

**Background:**

Many natural products have immunomodulatory properties. However, the mechanism of immunomodulatory activities are poorly understood.

**Objectives:**

This study evaluated the influence of bovine colostrum products, a whey product, or their combinations with other natural products on human peripheral blood mononuclear cells’ (PBMC) ability to produce cytokines upon activation.

**Methods:**

PBMCs were pretreated with ultrafiltered colostrum, nano-filtered bovine colostrum, egg yolk extract, a botanical blend, colostrum + egg yolk extract, colostrum + egg yolk + botanical blend, and fermented whey and then stimulated with lipopolysaccharide or phytohemagglutinin. Cytokine production was measured by the Luminex assay.

**Results:**

All study products demonstrated immunomodulatory properties by regulating cytokines production by activated PBMCs. Ultrafiltered colostrum alone displayed the highest immune stimulatory activity. It stimulated proinflammatory cytokine production by lipopolysaccharide-activated PBMCs and suppressed cytokine production by phytohemagglutinin-activated cells. Other study products mainly suppressed cytokine release by both cell types. The immunomodulatory properties depended upon the dose of the products used in the study.

**Conclusions:**

All tested products modulated innate and adaptive immune cell activities. Most of the products demonstrated anti-inflammatory properties, except ultrafiltered colostrum, which stimulated the lipopolysaccharide-activated PBMC production of inflammatory cytokines. These products can be potentially used to support overall immune health.

## Highlights

• Folk medicine used natural products for the prophylaxis and treatment of infectious diseases for centuries. A few decades ago, we learned that many natural products have immunomodulatory properties. However, the mechanism of immunomodulatory activities was not clear.
• In this study, we demonstrate that tested natural product blends modulate the activity of both cells of innate and acquired arms of the immune system. Most of the studied products suppressed the production of inflammatory cytokines by activated PBMCs. The ultrafiltrate colostrum, however, stimulated the production of inflammatory cytokines by LPS-activated PBMCs and suppressed their production by PHA-activated cells.

## Introduction

The immune system's primary function is the protection of an organism from infectious agents.^1^ The immune system is also involved in maintaining tissue integrity, wound repair, and supporting and regulating the metabolism and functions of different organs and systems.^2^ Therefore, support of an adequately balanced immune system function is crucial for maintaining the well-being of people.

Several studies have investigated how different compounds, such as vitamins, minerals, medicinal herbs, and proteins, can influence immune system functions.^3^, ^4^, ^5^ Zinc deficiency, for example, is associated with a reduction of the functions of several immune cell types, including natural killer (NK) cells and T cells.^6^ Vitamin D3 stimulates the differentiation of macrophages their bactericidal activity, decreases T helper (Th) 1 cell (inflammatory) activity, and increases the activity of Th2 (anti-inflammatory) cells.^7^ Beta-glucans, found in mushrooms, can activate the complement system by enhancing NK cells and macrophage activity.^8^ Bovine colostrum, comprising immunoglobulins, antimicrobial peptides, and several other bioactive molecules, modulates both arms of the immune system (eg, B cells, Th cells, regulatory T cells, NK cells, and macrophages) and may help the host resist microbial infections.^9^

Although many studies describing the effects of natural products on the immune system have been published, there is no or very little research investigating the influence of the combinations of these products or products that undergo manufacturing changes (eg, defatting or enrichment of bioactive compounds of interest) on the immune system.

Bovine colostrum and whey are 2 popular natural products that often undergo manufacturing changes to optimize their immune health benefits. Bovine colostrum may be defatted and ultrafiltered to concentrate the smaller peptides and proteins.^10^ Whey is usually hydrolyzed and enzymatically digested into bioactive peptides.^11^ Previous studies showed that both ultrafiltered bovine colostrum and hydrolyzed whey increase NK cell activity and the production of interleukin (IL) 2, interferon (IFN) γ, and tumor necrosis factor alpha (TNFα) by NK cells after their oral administration to mice.^12^, ^13^, ^14^

The study presented herein evaluates the effects of colostrum-based natural products, whey extract, and their combinations with egg yolk extract and mushroom extracts on the ability to modulate the immune activity of peripheral blood mononuclear cells.

## Materials and Methods

### Human peripheral blood mononuclear cells

Human Peripheral Blood Leukopak was purchased from StemCell Technologies (Vancouver, British Columbia, Canada). The protocol and donor consent form for blood cell collection was approved by our facilities institutional review board.^15^ The typical Leukopak contains ∼27% CD4+ T cells, ∼16 CD8+ T cells, 8.5% B cells, and ∼26% mono- and granulocytes.^15^ Collected cells were free of bloodborne infections. The human peripheral blood mononuclear cells (PBMCs) were isolated using the Ficoll gradient procedure,^16^ washed, and then cryopreserved.

### Study products

The study products and their bioactive components, brand names, and product lots used in the study are listed in Table 1. All products are supplied by 4Life Research USA (Sandy, Utah). Ultra-filtered colostrum was prepared by defatting whole bovine colostrum and ultrafiltering to concentrate the peptides and proteins with molecular sizes smaller than 10,000 Daltons. Nano-filtered colostrum comprises proteins and peptides with molecular weights of less than 5000 Daltons. Chicken egg yolk extract was prepared by spray-drying the egg yolk. The botanical blend is composed of soy phytosterols, lemon peel powder, and the extracts of *Grifola frondosa, Lentinus edodes, Cordyceps sinensis, Agaricus blazeii*, baker's yeast, aloe gel, oat seed, and olive leaf. The combined product of colostrum, egg yolk extract, and botanical bland is composed of ultrafiltered colostrum and egg yolk extract (>20%), soy phytosterols (>20%), inositol hexaphosphate (>15%), *Cordyceps sinensis* mycelia extract (>5%), lemon peel powder (<5%), *Grifola frondose* fruiting body extract (<5%)*, Lentinus edodes* fruiting body extract standardized (<5%), *Agaricus blazeii* fruiting body extract (<5%), baker's yeast standardized to 3% polyphenols (<5%), aloe vera (*Aloe barbadensis*) leaf gel (<5%), oat seed extract (<5%), and olive (*Olea europae*) leaf (<5%).

**Table 1:** Study products used for PBMC treatment.

Study product name⁎	Bioactive components of products	Brand name	Lot No. of products used in the study
Ultrafiltered colostrum	Peptides and proteins (≥37%) and maltodextrin (<5%)	Transfer Factor Classic	B2025806
Nanofiltered colostrum	Proteins and peptides with MW 5,000 Daltons (≥37%) and maltodextrin (<5%)	NanoFactor	B2020314
Egg yolk extract	Proteins and peptides (≥37%)	OvoFactor	B2030008
Botanical blend	Soy phytosterols, inositol hexaphosphate, *Cordyceps sinensis mycelia* extract standardized to 10% cordycepic acid, lemon peel powder, *Grifola frondose* fruiting body extract standardized to 10% D-Fraction, *Lentinus edodes* fruiting body extract standardized to 10% D-Fraction, *Agaricus blazeii* fruiting body extract standardized to 10% polysaccharides, baker's yeast standardized to 3% polyphenols, aloe vera (*Aloe barbadensis*) leaf gel standardized to 5% polysaccharides, oat seed extract standardized to 5% beta-glucan, and olive (*Olea europae*) leaf standardized to 20% oleuropein.	Cordyvant Complex	AA11302020-01
Colostrum + egg	Peptides and proteins, including immunoglobulins (>37%) and maltodextrin (<5%), and silicon dioxide	Transfer Factor Tri-Factor	20050132
Colostrum +egg + botanical	Ultrafiltered colostrum and egg yolk (>20%), soy phytosterols (>20%), inositol hexaphosphate (>15%), Cordyceps sinensis mycelia extract standardized to 10% cordycepic acid (>5%), lemon peel powder (<5%), *Grifola frondose* fruiting body extract standardized to 10% D-Fraction (<5%), *Lentinus edodes* fruiting body extract standardized to 10% D-Fractio (<5%), *Agaricus blazeii* fruiting body extract standardized to 10% polysaccharides (<5%), baker yeast standardized to 3% polyphenols (<5%), aloe vera (*Aloe barbadensis*) leaf gel standardized to 5% polysaccharides (<5%), oat seed extract standardized to 5% beta-glucan (<5%), and olive (*Olea europae*) leaf standardized to 20% oleuropein (<5%).	Transfer Factor Plus	AA11302020-02
Fermented whey	Enzyme-cleaved sweet whey (≥90% peptides and proteins)	Whey Factor	2558321

⁎ All study products used in this study were produced by 4Life Research, LLC. (Sandy, UT, USA).

Study products were solubilized in dimethyl sulfoxide (DMSO) and further diluted to the assay concentrations with a complete medium (CM) containing RPMI 1640, 10% heat-inactivated fetal bovine serum, 1% penicillin/streptomycin, and 2 mM L-glutamine. The final concentration of DMSO in the culture was 0.1%.

### Cell viability assay

AlamarBlue reagent at a 1:10 dilution was added to PBMCs treated with lipopolysaccharide (LPS) or phytohemagglutinin (PHA), or the test compounds plus LPS or PHA (see details below). Because the test products were diluted in DMSO, AlamarBlue reagent was also added to the wells of untreated cells containing 0.1% DMSO to establish baseline cell viability (baseline control). After 4-hour incubation, plates were read on a fluorescence plate reader at 530 nM (530–570 nM filter) for excitation and 595 nM (580–610 nM filter) for emission. Relative fluorescence units were measured. Cell viability was presented as a percentage of control values using the following equation:

$$\frac{\text{Mean}\;\text{relative}\;\text{fluorescence}\;\text{value}\;\text{of}\;\text{test}\;\text{sample}\;\times \;100}{\text{Mean}\;\text{relative}\;\text{fluorescence}\;\text{of}\;\text{baseline}\;\text{control}}$$

### Cytokine Production by LPS-activated PBMCs

Cryopreserved human PBMCs were drip-thawed, washed in CM, and plated in triplicates at the cell density of 2 × 10^5^ cells/well in 150 µL CM into 96-well polystyrene plates (Corning, Glendale, Arizona). Study products were then added to the cells (10 µL) to the final concentrations of 2 µg/mL or 20 µg/mL and incubated in a humidified atmosphere at 37°C, 5% carbon dioxide for 1 hour. Some cells were cultured in the presence of dexamethasone (Dex), a broad-spectrum anti-inflammatory drug used as a reference control. Dex was added in a volume of 10 µL to a final concentration of 100 nM.^17^ After the initial 1-hour incubation, PBMCs were stimulated with LPS *Escherichia coli* O111:B4 (Millipore Sigma, Burlington, Massachusetts) at the final concentration of 50 pg/mL.^18^ LPS-activated PBMCs untreated with the products were used as a positive control. Plates were incubated in a humidified atmosphere at 37°C and 5% carbon dioxide for 24 hours. Then, plates were centrifuged at 200 g for 10 minutes; supernatants were collected and stored at –80°C for later Luminex multiplex cytokine analysis. (EMB Millipore Sigma, Burlington, MA, USA).

Luminex assessment of cytokine levels (human IL-1β, IL-6, and IL-8, and TNFα) in cell culture supernatants was performed per the manufacturer's protocol using the Human Cytokine/Chemokine Magnetic bead panel from EMD Millipore Sigma (St Louis, Missouri). The samples were diluted 1:8 based on the vendor-specified recommendations. The obtained cytokine values were multiplied by the dilution factor to obtain the final cytokine concentrations per milliliter of the supernatant.

### Cytokine Production by PHA-activated PBMCs

The PBMC culture was set up as described above. After the initial 1-hour incubation with the study products or Dex, PHA was added to the culture at a final 10 µg/mL concentration. PHA-activated PBMCs were used as a control. Cell culture supernatants were collected after 48 hours and stored at –80°C for later Luminex multiplex cytokine analysis.^19^^,^^20^ All samples were diluted based on the vendor-specified dilution recommendations (1:2 dilution for IL-1β, IL-5, IL-10, and IL-13; and 1:8 dilution for IFNγ, and TNFα). The obtained cytokine values were multiplied by the dilution factor to obtain the final cytokine concentrations per milliliter of the supernatant.

### Data analysis

Levels of cytokine in the supernatants were calculated as concentrations based on a 5-point standard curve, a nonlinear regression analysis. Outlier calculations were performed using the Grubbs' Maximum Normed Residual Test available in GraphPad Prism version 8.0 (GraphPad Inc, La Jolla, California). All data are presented as the average of 3 replicates (SD). Due to the moderate cytotoxicity of some test products, the cytokine levels were recalculated per 100,000 viable cells. Statistical differences were calculated by ANOVA test in GraphPad Prism version 8.0.

## Results

### Cell viability

The analysis of the cytotoxic effects of the study products and other chemicals used in the cell culture is present in Table 2. When the cytotoxicity of a compound is evaluated, the results of cytotoxic assay need to meet all following criteria: “the signal in the cells treated with the test substance is reduced at least by 20% compared with the negative controls,” “a concentration-related reduced signal is observed,” and the results are reproducible.^21^ It has been shown that DMSO at concentrations higher than 1% can influence cell viability.^22^ The final DMSO concentration in our experiment was 0.1%. The addition of DMSO did not influence cell viability compared with naive cells (data not shown). The activation of PBMCs with LPS resulted in a reduction of cell viability by ∼17%. The addition of study products to LPS-activated PBMCs did not result in a substantial reduction in cell viability compared with LPS-activated cells. The study products demonstrated a degree of cytotoxicity following incubation of PBMCs with PHA and the study products (Table 2). In some instances, the percent of dead cells was ≥30% compared with control. In this assay, the cytotoxicity of the test compounds was not dose-dependent, for example, ultrafiltered colostrum at concentration 2 µg/mL had 84% of viable cells and at concentration 20 µg/mL the viability of cells increased to 108% compared with control with the exception of egg yolk extract and botanical blend products. Due to the decreased cell viability, the cytokine production was calculated per 100,000 viable cells.

**Table 2:** Cell viability.

Treatment†	Cell viability*	
	+PHA, 10 µg/mL	+LPS, 50 pg/mL
Untreated cells + 0.1% DMSO	121 (9)	83 (25)
Dexamethasone, 100 nM	125 (9)	94 (8)
Fermented whey		
2 µg/mL	101 (490) (5)	83 (3)
20 µg/mL		84 (13)
Ultrafiltered colostrum		
2 µg/mL	84 (11) 108 (20)	88 (4) 96 (4)
20 µg/mL		
Nano-filtered colostrum		
2 µg/mL	83 (4)	89 (9)
20 µg/mL	79 (6)	77 (6)
Egg yolk extract		
2 µg/mL	81 (20)	86 (5)
20 µg/mL	72 (4)	90 (3)
Botanicals		
2 µg/mL	79 (20)	83 (9)
20 µg/mL	69 (16)	84 (11)
Ultra-nano colostrum + egg yolk extract		
2 µg/mL	82 (12) 130 (15)	85 (5)
20 µg/mL		88 (2)
Ultra-nano colostrum + egg yolk extract + botanicals		
2 µg/mL	87 (14)	85 (13)
20 µg/mL	81 (18) (19)	85 (7)

DMSO = dimethylsulfoxide; LPS = lipopolysaccharide; PHA = phytohemagglutinin.

⁎ Values are presented as mean (SD) mean is percent of viable cells. Cell viability is presented as the percentage of untreated control + 0.1% DMSO from triplicate measurements

† Cells were pretreated with the test compounds at 2 or 20 µg/mL, followed by activation with PHA 10 µg/mL for 48 hours or LPS 50 pg/ml for 24 hours.

### Cytokine production by LPS-activated PBMCs

**Figure 1:**
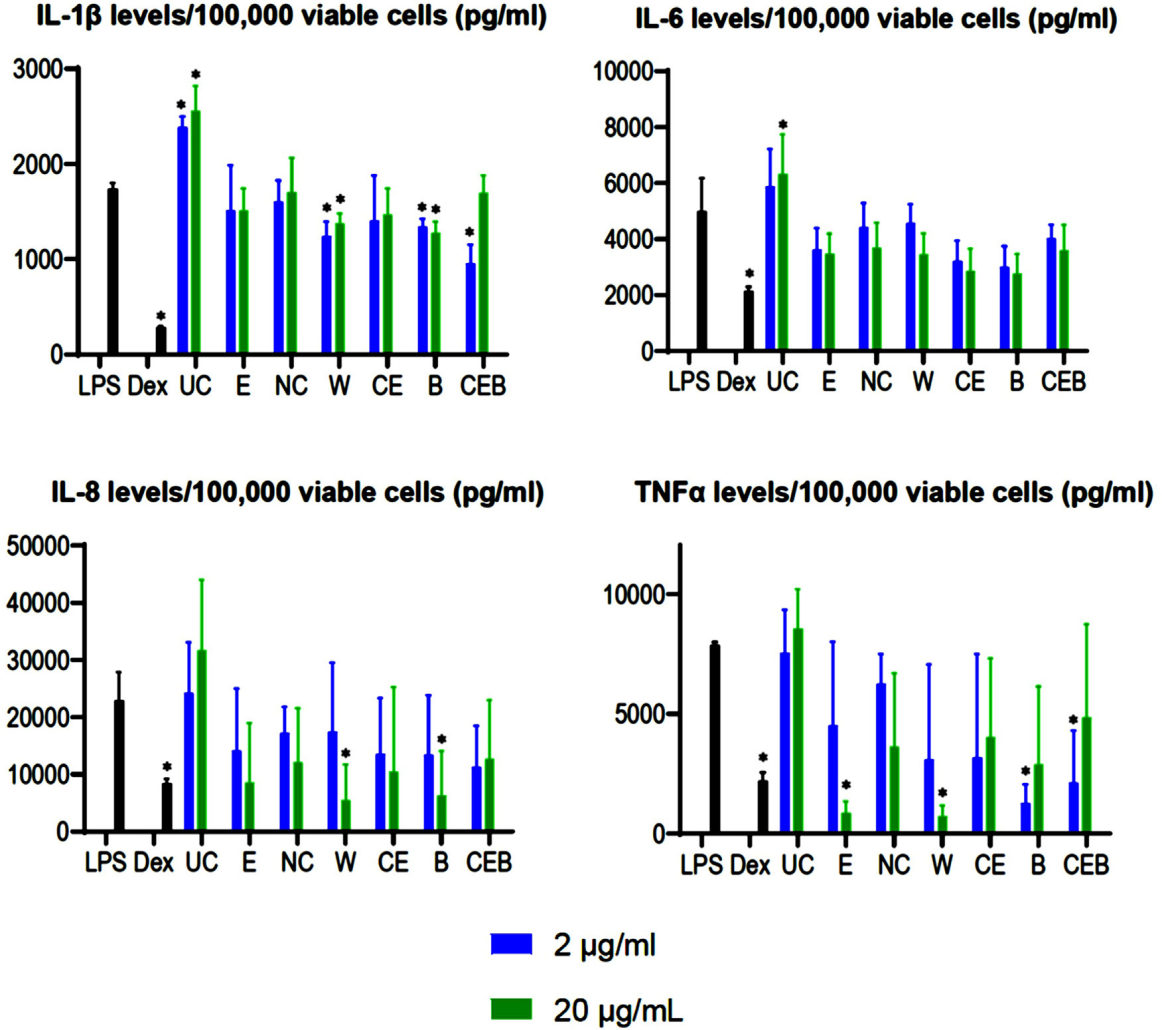
Cytokine production by lipopolysaccharide (LPS)-activated peripheral blood mononuclear cells (PBMCs). Black bars indicate treatment with LPS (50 pg/mL) or treatment with LPS plus dexamethasone (Dex) (100 nM); blue bars indicate treatment with 2 µg/mL study product; green bars indicate treatment with 20 µg/mL study product. All data are presented as the mean of 3 replicates (SD). B = botanical blend; CEB = ultra-nanofiltered colostrum + egg yolk extract + botanical blend; CE = ultra-nanofiltered colostrum + egg yolk extract; E = egg yolk extract; NC = nanofiltered colostrum; UC = ultrafiltered colostrum; W = fermented whey. *Cytokine responses that are significantly different (*P* ≤ 0.05) from that of the untreated LPS-stimulated PBMCs.

As expected, in the present study, LPS-activated PBMCs produced significant levels of cytokines (IL-1β, IL-6, IL-8, Macrophage Inflammatory Protein-1 alpha (MIP-1α), and TNFα) (Figure 1). The addition of Dex significantly inhibited cytokine production by LPS-activated PBMCs.

All study products modulated the activity of LPS-activated PBMCs (Figure 1). Unfiltered colostrum significantly increased the LPS-stimulated secretion of IL-1β when used at 2 and 20 µg/mL concentrations and interleukin 6 secretion at the concentration of 20 µg/mL (Figure 1). Ultrafiltered colostrum at the dose of 20 µg/mL stimulated the production of IL-8 and TNFα, but the differences between the production of these cytokines by PBMCs treated with ultrafiltered colostrum plus LPS and PBMCs treated with LPS were not statistically significant.

Egg yolk extract showed overall inhibitory effects on the ability of LPS-activated PBMCs to secret cytokines. (Figure 1). Differences were not statistically compared with the cytokine levels produced by LPS-only activated cells except for significant inhibition of TNFα production at the egg yolk concentration of 20 µg/mL.

When used at both concentrations, NC slightly inhibited the secretion of the cytokines (IL-6, IL-8, and TNFα) by LPS-activated PBMCs and did not influence IL-1β production (Figure 1). The difference between the group pretreated with NC and stimulated with LPS and the group of cells stimulated with LPS-only was not statistically significant.

Whey significantly inhibited the IL-1β by LPS-activated PBMCs when used at both concentrations and the secretions of IL-8 and TNFα at the concentration of 20 µg/mL (Figure 1).

Ultra-nano colostrum + egg yolk extract inhibited the secretion of all 5 cytokines by LPS-stimulated PBMCs (IL-1β, IL-6, IL-8, and TNFα) at both concentrations 2 and 20 µg/mL. However, these differences were not statistically significant (Figure 1).

Botanical blend demonstrated an overall inhibitory effect on the ability of LPS-stimulated cells to produce cytokines (Figure 1). The botanical blend significantly inhibited the secretion of IL-1β at both concentrations, 2 and 20 µg/mL, and TNFα when added at 2 µg/mL to the culture, and the secretion of IL-8 at the concentration 20 µg/mL.

Ultra-nano colostrum + egg yolk extract + botanicals showed a maximal inhibitory effect on the secretion of cytokines by LPS-stimulated PBMCs at 2 µg/mL (Figure 1). It significantly inhibited the secretion of IL-1β and TNFα when used at this concentration.

### Cytokine production by PHA-activated PBMCs

**Figure 2:**
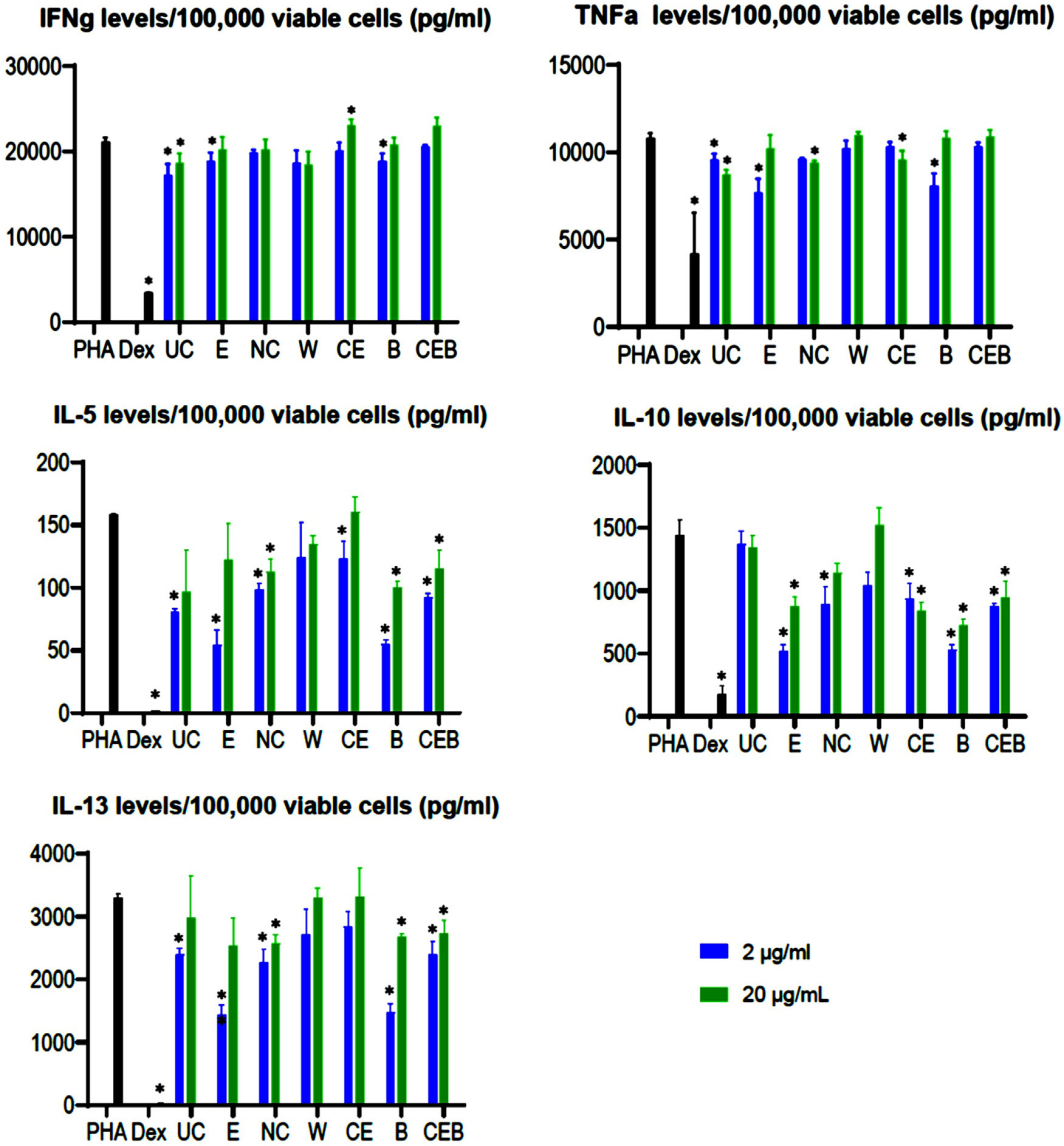
Cytokine production by phytohemagglutinin (PHA)-activated peripheral blood mononuclear cell (PBMCs) and treated with study products. Black bars indicate treatment with PHA (10 µg/mL) or treatment with PHA plus dexamethasone (Dex) (100 nM); blue bars indicate treatment with 2 µg/mL study product; green bars indicate treatment with 20 µg/mL study product. UC = ultrafiltered colostrum; E = egg yolk extract; NC = nanofiltered colostrum; W = fermented whey; CE = ultra-nanofiltered colostrum + egg yolk extract; B = botanical blend; CEB = ultra-nanofiltered colostrum + egg yolk extract + botanical blend. All data are presented as the mean of 3 replicates (SD). *Cytokine responses that are significantly different (*P* ≤ 0.05) from that of the untreated PHA-stimulated PBMCs.

PHA activation of PBMCs induced robust production of all tested cytokines. Dex treatment significantly inhibited PHA-induced cytokine production (Figure 2).

The treatment with ultrafiltered colostrum had an overall immunosuppressive effect on cytokine production by PHA-activated PBMCs (Figure 2). Ultrafiltered colostrum significantly inhibited PHA-induced IFNγ and TNFα production at both concentrations and IL-5 and IL-13 production at the concentration of 2 µg/mL.

Egg yolk showed overall inhibitory effects on all cytokines produced by PHA-activated PBMCs. Specifically, egg yolk significantly inhibited PHA-induced IFNγ, IL-5, IL-10, IL-13, and TNFα secretion at concentration 2 µg/mL and IL-10 production at concentration 20 µg/mL (Figure 2). Although egg yolk also inhibited IFNγ, IL-5, IL-13, and TNFα productions, the differences were not statistically significant.

The treatment with nano-colostrum reduced the production of all tested cytokines by PHA-activated PBMCs (Figure 2). Nano-colostrum significantly inhibited IL-5 and IL-13 production by PHA-activated PBMCs when used at concentrations 2 and 20 µg/mL, IL-10 at 2 µg/mL, and TNFα production when used at a concentration of 20 µg/mL only.

The treatment with fermented whey had a mixed effect on cytokine production by PHA-activated PBMCs (Figure 2). The treatment had no effect on TNFα production, reduced IFNγ, IL-5, and IL13 productions at both concentrations and stimulated IL-10 production at 20 µg/mL.

Similar to fermented whey treatment, colostrum + egg yolk treatment had a mixed effect on PHA-induced cytokine production by PBMCs (Figure 2). The treatment with colostrum + egg yolk significantly downregulated the production of IL-10 when used at 2 and 20 µg/mL concentrations and IL-5 production at 2 µg/mL concentrations only. IFNγ and IL-13 production were upregulated when PHA-activated cells were treated with 20 µg/mL colostrum + egg yolk.

The botanical blend had mainly immunosuppressive effects on cytokine production by PHA-activated PBMCs (Figure 2). The botanical blend significantly inhibited IL-5 and IL-13 production when used in both concentrations and IFNγ, IL-10, and TNFα when used at the concentration of 2 µg/mL only.

Colostrum + egg yolk + botanical blend significantly inhibited PHA-induced IL-5 and IL13 production at both concentrations, 2 and 20 µg/mL, and IL-10 when used at 20 µg/mL (Figure 2). Colostrum + egg yolk + botanical blend significantly had no effect on TNFα production and increased IFNγ secretion when used at 20 µg/mL.

## Discussion

The immunomodulatory properties of natural products are well known. In addition to modulating immune responses, many of these products also influence other organ and system functions. Several natural compounds (eg, flavonoid, nonflavonoid polyphenols, mycrosporine-like amino acids, and terpene) have ultraviolet light absorption properties and antioxidant and anti-inflammatory properties.^23^ *Moringa oleifera* extracts demonstrate antioxidant, hepatoprotective, radioprotective, and immunomodulatory activities.^24^ Glucans, the major bioactive compound in mushrooms, exhibit immunomodulatory and anticancer properties.^25^

In this study, we investigated the effects of multicomponent blends on the ability of LPS or PHA-activated PBMCs to produce an array of cytokines. Cytokines are small proteins produced by immune cells, usually upon cell activation. Cytokines facilitate communication between innate and adaptive arms of the immune system and between immune and nonimmune cells.^26^ It appears that study products had diverse effects on the ability of PBMCs to produce cytokines upon activation. Most of the studied products demonstrated anti-inflammatory properties.

In 1 set of experiments, PBMCs were activated with LPS. LPS is a component of the outer membrane of gram-negative bacteria that stimulates monocytes, macrophages, and B cells to produce inflammatory cytokines.^27^ Activated phagocytic cells release a specific set of cytokines that help to destroy pathogens or repair damage by inflamed tissues.^27^ Activation of B cells with LPS results in cell differentiation to antibody-producing cells.^28^ In vivo, LPS stimulation also results in T cell activation, but it is likely through its effect on antigen-presenting cells like dendritic cells or macrophages.^29^ Therefore, in our experiments, when PBMCs were activated by LPS, the cytokine levels detected in the supernatants resulted from the complex activation of monocytes, B cells, and potentially T cells.

Ultrafiltered colostrum showed a significant stimulatory effect on the ability of PBMCs to produce IL-1β and IL-6 when PBMCs were stimulated with LPS. IL-1β is a proinflammatory cytokine produced mainly by monocytes and macrophages at the early stages of infections.^26^ This cytokine stimulates the expansion and differentiation of CD4+ T cells. IL-6 may demonstrate anti-inflammatory and proinflammatory properties.^26^ IL-6 stimulates B cells' differentiation into plasma cells and cytotoxic T lymphocyte activity while suppressing regulatory T cells. Bovine colostrum has been shown to increase the number of CD14+ IL-12+ monocytes following PBMC treatment with IFNγ and LPS activation.^30^ The same study reported that bovine colostrum enhanced IFNγ secretion in response to weak antigenic stimulation by, for example, cytomegalovirus and inhibited IFNγ production in response to strong antigenic stimulation. This may suggest that bovine colostrum could exert its immunomodulatory effects by boosting the cytokine when an immune response is suboptimal and suppressing the same cytokine production when the immune system is hyperactivated. In our study, bovine colostrum enhanced the secretion of IL-10 and IL-2 while suppressing the secretion of IFNγ and TNFα when human PBMCs were stimulated with PHA (unpublished data). The composition of ultrafiltered colostrum is different from that of bovine colostrum due to processes of ultra- and nanofiltration. Following the ultrafiltration process, the final product contains bioactive products with molecular sizes smaller than 10,000 Daltons. Bovine colostrum contains immunoglobulins (secretory immunoglobulin A, M, and G) with molecular weight ranging from approximately 150,000 to 970,000 Daltons.^31^^,^^32^

Other tested products showed a suppressive effect on cytokines produced by LPS-activated PBMCs. The botanical blend and colostrum + egg yolk + botanical blend significantly suppressed productions of IL-1β and TNFα following treatment at low concentration (2 µg/mL). The botanical blend contains medicinal mushrooms *Grifolda frondosa, Lentinus edodes, Cordyceps sinensis*, and *Agaricus blazeii*. It has been reported that β-glucans isolated from *Grifolda frondose* or *Lentinus edodes* have anti-inflammatory properties.^33^ *Cordyceps sinensis* extracts may demonstrate either pro- or anti-inflammatory properties.^33^ The immunomodulatory properties of *Cordyceps sinensis* extracts depend on whether it is an aqueous or alkaline extract. The colostrum + egg yolk + botanical blend showed a similar to the botanical blend immunomodulatory profile, possibly due to the presence of mushroom extracts. Our data indicate that the test products (eg, ultrafiltered colostrum, colostrum + egg yolk, and colostrum + egg yolk + botanical blend) stimulate human NK cell activity and IFNγ and granzyme B productions (unpublished data).

PBMCs were activated by PHA in another set of experiments. Typically, T cells present in the peripheral blood respond to PHA activation.^34^ PHA is a potent mitogen that can induce the proliferation of T cells and cytokine secretion.^35^ It has been reported that PHA activates both Th cells and cytotoxic T cells.^36^^,^^37^ T cells are part of the adaptive arm of the immune system and play an essential role in shaping immune responses to microbial infections.^1^ Following microbial infection, dendritic cells process microbial antigens and present them as peptides in the context of Major Histocompatibility Complex (MHC) class II to Th cells. Th cells, upon activation, release cytokines and aid other immune cells recruited to the site of infection to eliminate the pathogens. The processed antigens presented to cytotoxic T cells activate these cells and stimulate their cytotoxic activity against pathogen-infected cells.

The study products significantly influenced the adaptive arm of the immune system, evidenced by changes in the levels of cytokines produced by PHA-activated PBMCs. The treatment with ultrafiltered colostrum inhibited PHA-stimulated PBMC production of IFNγ, IL-5, IL-10, IL-13, and TNFα. IL-5 is produced by Th cells and is a potent activator of eosinophils.^38^ IL-5 is critical in the pathogenesis of asthma, eosinophilic granulomatosis, and eosinophilic chronic rhinosinusitis.^38^ Anti–IL-5 monoclonal antibodies are proven effective in treating these diseases; therefore, suppression of IL-5 production by ultrafiltered colostrum may ameliorate eosinophil-mediated conditions. IFNγ regulates immune responses against intracellular microorganisms and controls tumor immunity.^39^ NK and T cells are the primary producers of IFNγ.^39^ It appears that murine NK cells are the targets of ultrafiltered colostrum and fermented whey.^13^ TNFα is among the inflammatory cytokines regulating cell proliferation and survival, leukocyte adhesion, blood coagulation, and some metabolic processes.^40^ Dysregulated signaling through TNF receptor 1 (TNFR1) and the production of TNFα may lead to the development of rheumatoid arthritis, inflammatory bowel disease, and psoriasis.^41^

Nano-colostrum significantly inhibited IL-5 and IL-13 production by PHA-stimulated PBMCs. IL-13 regulates the differentiation of alternative (M2) macrophages.^42^ Similar to IL-5, IL-13 is involved in modulations of allergic inflammatory responses. These findings suggest that Nano-colostrum may help support sinus health in people prone to allergic reactions.

The botanical blend, whey protein, and other natural products' combinations had mostly anti-inflammatory effects by suppressing inflammatory cytokines produced by PHA-activated PBMCs. Mushrooms are part of the botanical blend. Amino acids present in mushrooms are mainly responsible for their anti-inflammatory properties via modulating prostaglandin metabolism.^43^ Our findings are in good agreement with previously published data that whey proteins decrease inflammatory cytokine production via suppression of nuclear factor kappa beta expression.^44^ Additionally, whey proteins may stimulate immunoglobulin G production via modulation of Th cell functions.^45^ The review of preclinical and clinical trial results conducted by Hetland et al^46^ showed that medicinal mushrooms possess anticancer and antiallergenic properties in addition to anti-inflammatory properties. It appears that mushrooms' anticancer and antiallergenic properties are exerted mainly via modulation of T cell functions.

### Study limitations

The results of this in vitro study cannot be generalized to humans. More studies are needed to understand how the products modulate innate and adaptive immune responses. Clinical trials are necessary to confirm the observed immunomodulatory effects in humans.

## Conclusions

Most of the study products demonstrated anti-inflammatory properties suppressing the production of cytokine by either LPS or PHA-activated PBMCs. The products’ immunomodulatory properties depended on the PBMC pretreatment dose. An outstanding product is ultrafiltered colostrum. It stimulated cytokine production by LPS-activated PBMCs (likely monocytes and B cells) and suppressed the production of cytokines by PHA-activated PBMCs (likely T cells). The study presented evidence that these products might be used to support overall immune health.

## Declaration of Competing Interest

This study was funded by 4Life Research, LLC (Sandy, Utah).

The authors have indicated that they have no other conflicts of interest regarding the content of this article.
